# Interest of Pet Imaging in Multiple Myeloma

**DOI:** 10.3389/fmed.2019.00069

**Published:** 2019-04-09

**Authors:** Bastien Jamet, Clément Bailly, Thomas Carlier, Cyrille Touzeau, Cristina Nanni, Elena Zamagni, Louisa Barré, Anne-Victoire Michaud, Michel Chérel, Philippe Moreau, Caroline Bodet-Milin, Françoise Kraeber-Bodéré

**Affiliations:** ^1^Nuclear Medicine Unit, University Hospital, Nantes, France; ^2^CRCINA, INSERM, CNRS, Nantes University, Nantes, France; ^3^Haematology Department, University Hospital, Nantes, France; ^4^Nuclear Medicine, Azienda Ospedaliero-Universitaria di Bologna, Bologna, Italy; ^5^Seràgnoli Institute of Hematology, Bologna University School of Medicine, Bologna, Italy; ^6^Unicaen-CEA-CNRS, UMR6030, Caen, France; ^7^Nuclear Medicine Unit, ICO-Gauducheau, Nantes-Saint-Herblain, France

**Keywords:** multiple myeloma, PET/CT imaging, FDG-PET/CT, review, prognosis

## Abstract

The interest of 18Fluoro-deoxyglucose (FDG) positron emission tomography (PET) imaging in the management of patients with multiple myeloma (MM) for the workup at diagnosis and for therapeutic evaluation has recently been demonstrated. FDG-PET is a powerful imaging tool for bone lesions detection at initial diagnosis with high sensitivity and specificity values. The independent pejorative prognostic value on progression-free survival (PFS) and overall survival (OS) of baseline PET-derived parameters (presence of extra-medullary disease (EMD), number of focal bone lesions (FLs), and maximum standardized uptake values [SUV_max_]) has been reported in several large independent prospective studies. During therapeutic evaluation, FDG-PET is considered as the reference imaging technique, because it can be performed much earlier than MRI which lacks specificity. Persistence of significant FDG uptake after treatment, notably before maintenance therapy, is an independent pejorative prognostic factor, especially for patients with a complete biological response. So FDG-PET and medullary flow cytometry are complementary tools for detection of minimal residual disease before maintenance therapy. However, the definition of PET metabolic complete response should be standardized. In patients with smoldering multiple myeloma, the presence of at least one hyper-metabolic lytic lesions on FDG-PET may be considered as a criterion for initiating therapy. FDG-PET is also indicated for initial staging of a solitary plasmacytoma so as to not disregard other bone or extra-medullary localizations. Development of nuclear medicine offer new perspectives for MM imaging. Recent PET tracers are willing to overcome limitations of FDG. (11)C-Methionine, which uptake reflects the increased protein synthesis of malignant cells seems to correlate well with bone marrow infiltration. Lipid tracers, such as Choline or acetate, and some peptide tracers, such as (68) Ga-Pentixafor, that targets CXCR4 (chemokine receptor-4, which is often expressed with high density by myeloma cells), are other promising PET ligands. 18F-fludarabine and immuno-PET targeting CD138 and CD38 also showed promising results in preclinical models.

## Introduction

Multiple myeloma (MM) is a hematological neoplasm characterized by the clonal proliferation of malignant plasma cells in the bone marrow. It is almost always preceded by an initial monoclonal gammopathy of undetermined significance (MGUS), that then develops into asymptomatic or Smoldering MM (SMM), which constitutes an intermediate clinical stage between MGUS and MM.

The rate of progression from MGUS to MM is 0.5–1% per year, and that of SMM to MM 10% per year for the first 5 years, with the thresholds of serum M protein and spinal plasmacytosis differing between both classifications. SMM is a heterogeneous classification including patients with a very slow progression to proven MM (several years) and patients progressing very rapidly to symptomatic MM in <2 years (high-risk SMM). The definition of symptomatic MM, a clinical stage requiring treatment, typically based on the presence of CRAB criteria (HyperCalcemia, Renal failure, Anemia, and Bone disease) (1) was revised in 2014 by the International Myeloma Working Group (IMWG) by integrating new prognostic biomarkers (2), with the aim of not delaying the initiation of treatment for patients classified as high risk SMM and to avoid progression to harmful bone lesions or renal insufficiency. Indeed, medullary plasmacytosis ≥60%, serum free light chain ratio ≥100 and more than 1 focal MRI bone lesion were predictive of an 80% progression to a CRAB-positive MM within 2 years in several studies, confirming a stage of the disease requiring treatment.

In addition, the 2014 IMWG criteria for the diagnosis of MM highlighted the importance of new imaging in the management of MM in order to detect bone disease, which is considered as a symptomatic MM criterion requiring treatment even when asymptomatic. Studies conducted over the past 10 years have shown better performance using low-dose whole-body CT and MRI scans (3, 4) than standard skeletal radiographs, formerly considered as the reference technique for detecting bone disease.

Recent data suggest that positron emission tomography (PET) using ^18^F-deoxyglucose (FDG) is a reliable imaging for initial staging, therapeutic monitoring and relapse workup in MM, especially because of its prognostic potential (5). Moreover, as shown recently in a prospective comparison between size of biopsied focal bone lesions (FL) depicted by FDG-PET and genomic profiles, the extent of spatial heterogeneity is positively associated with the size of FL, resulting coexistence of different disease clones (6). More recent PET tracers (Methionine, lipid and peptide tracers) are available to overcome limitations of FDG.

## Performance of FDG-PET for the Detection of Medullary and Extra-Medullary Disease at Initial Diagnosis

**Figure 1:**
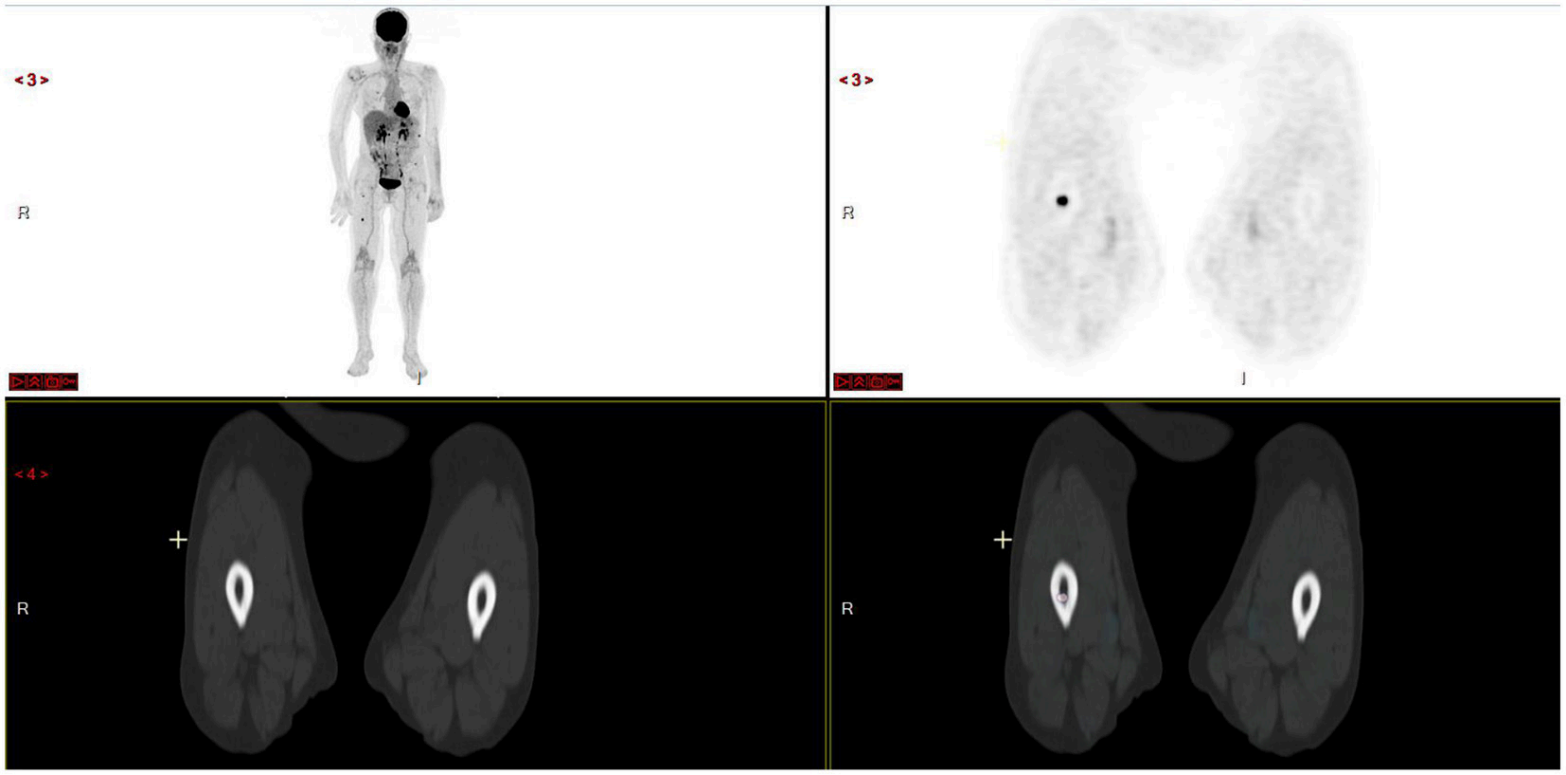
Patient with more than 10 focal lesions of the axial and appendicular skeleton. Note absence of osteolysis on opposite CT scan of right femur focal lesion.

**Figure 2:**
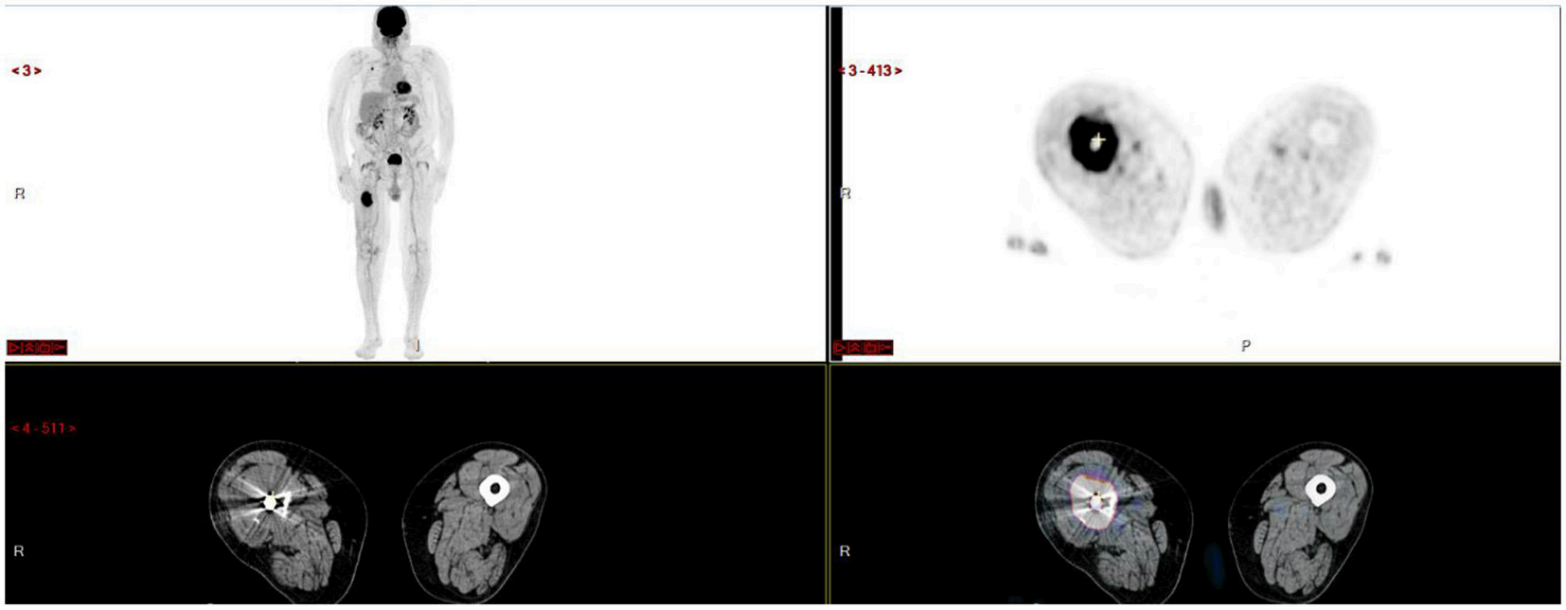
Voluminous hypermetabolic lytic lesion of the right femoral shaft contiguously invading adjacent soft tissues.

**Figure 3:**
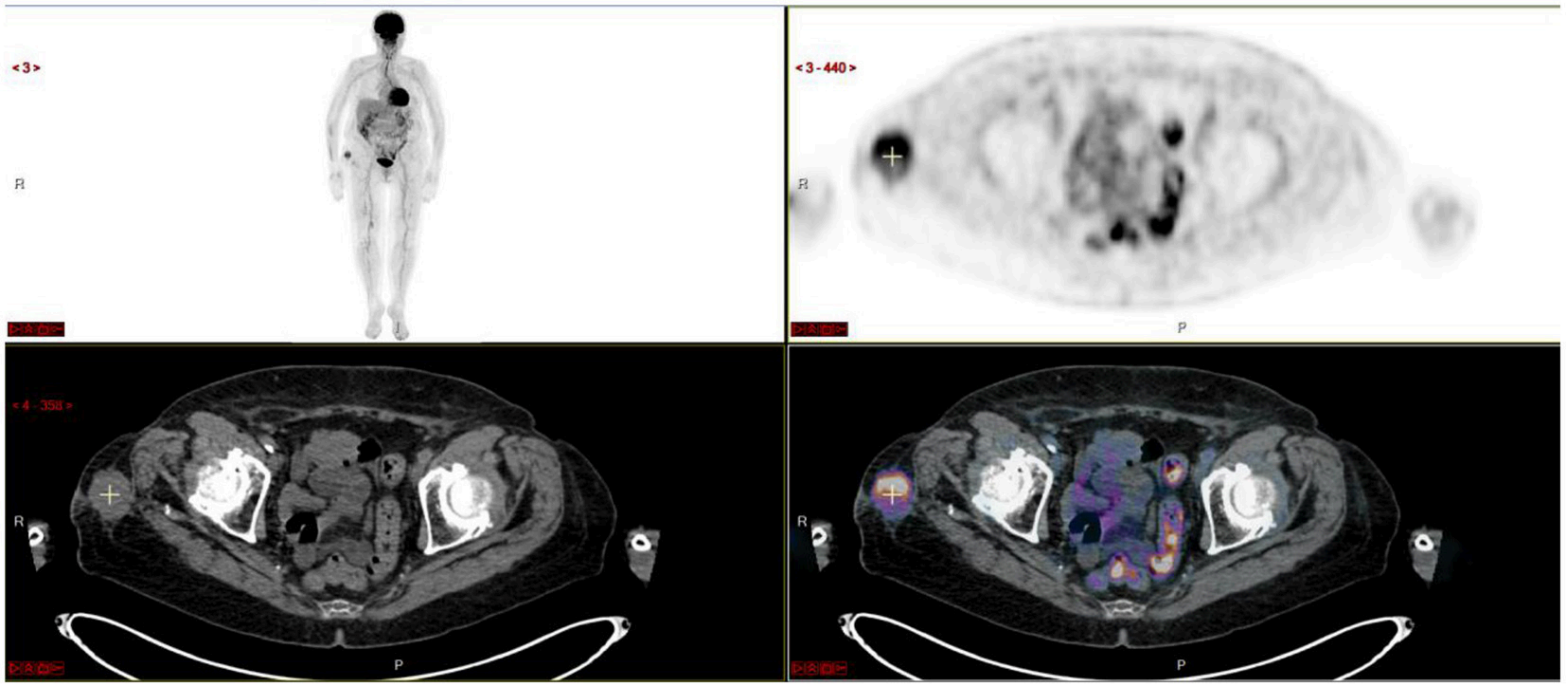
Extra-medullary disease histologically proven in this subcutaneous mass.

**Figure 4:**
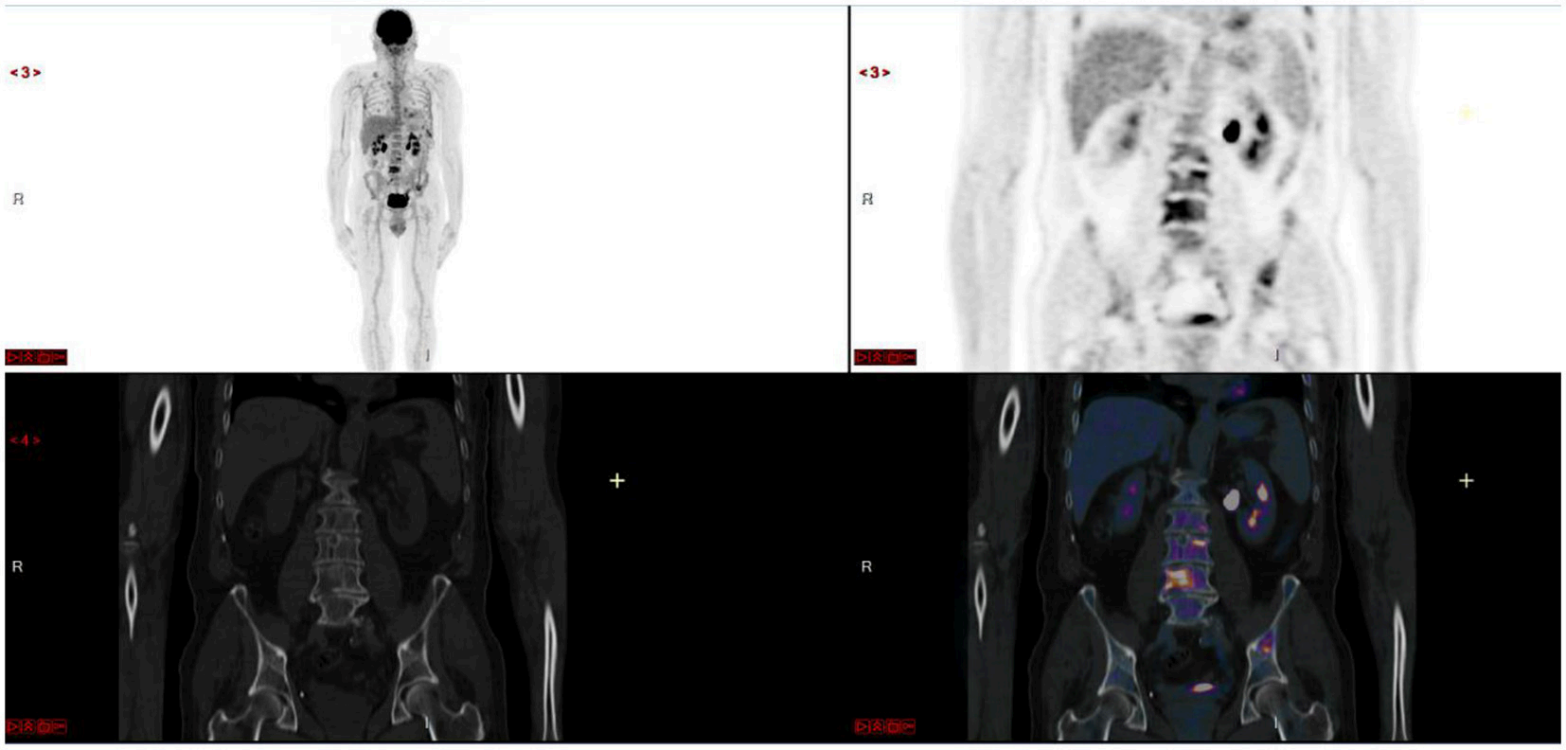
Diffuse medullary involvement (and superimposed lesions).

PET-FDG allows whole-body exploration and has a global sensitivity of 90% for the detection of medullary disease with a specificity varying from 70 to 100% according to several studies (7–9). Medullary abnormalities detected by PET are focal lesions (Figure 1), para-medullary lesions (PML, Figure 2) and diffuse medullary involvement with variable glucose uptake, resulting in variable SUV_max_ values (5–13). FDG-PET also allows the detection of extra-medullary disease (EMD, Figure 3), in <10% of patients at diagnosis (14). FL are most often defined as foci of uptake above the surrounding background noise on two successive sections with or without osteolysis opposite the CT image. PML are soft tissue invasions with contiguous bone involvement. Diffuse bone marrow involvement is usually defined as heterogeneous or homogenous diffuse uptake of the axial (that may extend to the peripheral) skeleton, of greater intensity than the liver (Figure 4). MM related disease abnormalities to be incorporated in the baseline FDG-PET report are presented in the Table 1.

**Table 1:** What should be provided in the FDG-PET report at baseline?

**MM related disease abnormalities**
**Focal Lesion (FL):** With or without osteolysis on CT: Total Number: 0, 1–3, >3; Intensity: Hottest SUV_max_ (Most intense FDG uptake identified among all foci determined by the nuclear physician); **Bone Marrow diffuse involvement (BMI):** Evaluation of the bone marrow diffuse uptake, regardless focal lesions: Visual analysis (Deauville 5-level scale): Positive if diffuse uptake of the axial (that may extend to the peripheral) skeleton is greater intensity than the liver (Deauville 4 or 5). **Extramedullary disease (EMD):** Soft tissue or nodal mass not directly adjacent to MM bone localization: Presence or not and localization. **Paramedullary disease (PMD):** Bone lesion involving surrounding soft tissues with bone cortical interruption: Presence or not, localization, clinical risk?

*MM, multiple myeloma; CT, computed tomography; SUV, standardized uptake value*.

The Bologna group recently proposed the “IMPETUS” criteria (15) to standardize the interpretation of PET in MM. It showed that the use of a standardized visual scale of interpretation (Deauville 5-level scale) in the description of the number of FL, EMD as well as diffuse medullary involvement makes it possible to improve the reproducibility of inter-observer interpretation (with however, a very great disparity in interpretation of skull lesions). The pathological positivity cut-offs for bone lesions, especially on therapeutic evaluation examinations, are still to be determined however, especially when comparing with sensitive biological techniques (CMF) for detection of MRD.

The sensitivity of FDG-PET is greater than whole-body radiology to detect bone lesions and comparable to or less than that of pelvic-spinal MRI (7, 12, 16–19). In the first small series of patients comparing FDG-PET and MRI, sensitivity of FDG-PET was less than that of pelvic-spinal MRI (PR-MRI) for diffuse medullary involvement but allowed detection of additional FL, especially outside the field of the MRI view (17). More recently, the French Imajem study (14) prospectively compared PR-MRI and FDG-PET at initial diagnosis and after therapy. In this cohort of 134 patients with symptomatic MM, PR-MRI was positive in 94.7% of cases and FDG-PET in 91% of cases, revealing an equivalent detection sensitivity.

FDG-PET also demonstrated interest in patients with solitary plasmacytoma (SP), allowing detection of additional lesions, with sensitivity, and specificity greater than MRI (5–7, 20). In addition, Fouquet et al. showed that the presence of at least 2 hypermetabolic lesions by FDG-PET was predictive of rapid progression to MM (21).

According to recent update data of the Southeastern Minnesota cohort (22) with a long-term follow-up, there are adversely risk factors for MGUS to active MM progression including an M-protein of 15 g/L or more and an abnormal free light chain ratio in patients with non-IgM MGUS. Patients with 2 risk factors showed a significantly higher progression rate to MM of 30% in 20 years than patients with no (7%) or 1 risk factor (20%). Therefore, there is a need of imaging for patients with high risk MGUS. To date, there are unfortunately no published data on FDG-PET findings in MGUS patients.

## Prognostic Value of FDG-PET in SMM and Symptomatic MM at Baseline Evaluation

FDG-PET showed prognostic value in patients with SMM and symptomatic MM.

Even if the latest international recommendations of the IMWG (1) indicate that the presence of one or more FL with osteolysis on FDG-PET is considered a criterion for treatment at initial diagnosis, all prospective studies lead from 2009 defined FL as foci of uptake with or without osteolysis cause metabolic could precede morphological abnormalities.

Moreover, in SMM, a positive FDG-PET defined by the presence of FL without underlying osteolytic lesions is associated with a rapid progression to symptomatic MM. Indeed, in a cohort of 122 SMM patients, Siontis et al. (23) showed that the probability of progression to MM within 2 years for positive FDG-PET patients was 75 vs. 30% for patients with a negative PET, without therapy. In another prospective study of 120 SMM patients, the group of Bologna (24) reported a rate of progression to symptomatic MM at 2 years of 58% for patients with positive PET vs. 33% for patients with a negative PET.

In symptomatic MM baseline evaluation, three large prospective studies have demonstrated important prognostic impact of FDG-PET results, which is particularly important at age of precision medicine and risk-based therapies. First of all, Bartel et al. in a large cohort (*n* = 239) treated using the Total Therapy 3 strategy (25) showed that the only imaging examination (between FDG-PET and MRI) significantly associated with an adverse prognosis for both overall survival (OS) and event-free survival (EFS) was FDG-PET when the number of FL was >3. Then, the Bologna group, in a large series of 192 MM patients also enrolled in a double autologous stem cell transplantation (ASCT) program after induction (26), confirmed the pejorative prognostic impact of more than 3 FL on progression-free survival (PFS) at 4 years as well as an SUV > 4.2 and the presence of EMD. SUV > 4.2 and the presence of EMD were also associated with a shorter OS. The prognostic value of EMD on PFS and OS was recently confirmed by the French Imajem study (14).

Two large retrospective studies found equal results about prognostic value of FDG-PET in symptomatic MM baseline evaluation. The Mayo Clinic team, in a 313 patient cohort showed that the presence of at least 3 FL and EMD predicted inferior OS (only by univariate analysis), with no clear SUVmax cutoff predictive of PFS or OS (27). In a smaller series of patients (*n* = 167), Jung et al. (28) confirmed (in multivariate analyses) that presence of more than three FL or EMD was associated with significantly inferior PFS and OS, especially in Revised International Staging System (R-ISS) II and III subgroups of patients.

More complex PET biomarkers, such as functional volumes and tumor heterogeneity, have also been studied or are being evaluated with promising results. First pre-therapeutic assessment of the whole-body total metabolic volume of FL and EMD (MTVWB) in 47 patients showed a poor prognostic value of high values on PFS and OS (29), with best discriminant cut-offs of 42.2 cm^3^ for the PFS and 77.6 cm^3^ for the OS. A second larger study of 192 patients confirmed the poor prognostic value of a high MTVWB, which was also similar for a high Total lesion glycolysis (TLG) WB (30). Indeed, by multivariate analysis, TLGWB > 620 g or MTVWB > 210 cm^3^ at baseline significantly decreased PFS and OS after adjustment for known prognostic factors. Combined with the gene expression profiling prognostic score (GEP70), a TLGWB > 205 g identified a high-risk subgroup and separated ISS II patients into two subgroups, with a similar outcome to ISS I and ISS III patients.

Finally, as described by Carlier et al. (31) for 66 patients of the Imajem study, intra-tumoral textural features (e.g., reflecting of tumor heterogeneity), especially energy, also seem to be of prognostic value (independent prognostic value of energy on PFS and OS). More work is in progress on this subject.

## Prognostic Value of FDG-PET in Therapeutic Evaluation

FDG-PET is considered as the reference imaging technique for therapeutic evaluation in MM with a strong independent prognostic value (5). FDG-PET allows evaluation of the response earlier than standard MRI but new MRI functional approaches, such as diffusion weighted imaging (DWI) measuring the apparent diffusion coefficient (ADC) influenced by tissue microarchitecture and related to marrow cellularity could be interesting tools to evaluate the disease after therapy (32, 33). However, homogeneous and prospective data about comparison between FDG-PET and WB-DWIMRI are lacking (34).

FDG-PET, coupled with a biological technique for the detection of minimal residual disease (MRD), makes it possible to improve the definition of complete response (35) clearly correlated with long-term outcomes.

All large prospective studies above mentioned have demonstrated the strong and independent prognostic impact of FDG-PET results after therapy of symptomatic MM.

The Little Rock team first showed in 2009 that normalization of FDG uptake of FL after chemotherapy induction cycles (before the transplant procedure) was associated with better EFS and OS (25). The same team reported in 2013 in a larger series of 302 patients (277 of them were also the object of a gene expression profile study) (36) treated according to the same intensive protocol that 3 FL on FDG-PET performed at Day 7 of induction was associated with lower PFS and OS, even in the high-risk group in relation to genetic profiling. FDG-PET could be considered as a tool for early therapeutic adaptation. They finally confirmed these results in 2018 from data collected in their TT4–TT6 clinical trials, in a very large cohort of more than 500 patients, showing patients achieving 100% suppression of FL signal following treatment at each time point studied (day 7, end of induction, post transplantation, and maintenance) had PFS and OS values that were not significantly different from cases with no FL present at baseline (37).

The Bologna group then showed that after induction therapy, a SUV > 4.2 was associated with a reduced PFS (26). Three months after ASCT, complete metabolic response (CMR) was achieved in 65% of patients, with PFS and OS at 4 years higher than those in PET-positive patients. Interestingly, 23% of patients achieving CR in accordance with conventional criteria were considered PET-positive. Multivariate analysis showed that post ASCT PET status was an independent prognostic factor of PFS. In 2015, the same group confirmed these results in 282 patients undergoing front line treatment between 2002 and 2012 (38). After treatment, a CMR was obtained in 70% of patients, whereas the conventional biological methods concluded at 53% of CR. The FDG-PET negativity affected the PFS and the OS positively.

The Imajem study more recently confirmed the major benefit of FDG-PET in therapeutic evaluation (14). Whereas, normalization of MRI after three cycle of combined induction therapy or before maintenance did not significantly affect either PFS or OS, FDG-PET normalization before maintenance was strongly associated with better PFS and OS. The PFS and OS of PET-negative patients were better than those of PET-positive patients (24-months PFS by 72 vs. 56.8%: *p* = 0.01; OS at 24 months of 94.2 vs. 72.9%: *p* = 0.03). In addition, multivariate analysis revealed that normalization of pre-maintenance FDG-PET was independently associated with longer PFS, such as absence of EMD at diagnosis and at least a very good partial biological response after three cycles of induction therapy.

Moreover, for the Imajem patients presenting a FDG-avid MM defined by lesion intensity higher than liver background, the prognostic value of FDG-PET after three cycles of induction therapy was also reported (39). Indeed, by multivariate analysis, only ΔSUVmax (*p* < 0.001) and biochemical response (*p* = 0.025) appeared as independent prognostic factors, with a more discriminative hazard ratio for ΔSUVmax analysis (>−25 vs. ≤−25%) which identified patients with improved median PFS.

The benefit of post-ASCT FDG-PET was also reported in 2013 in a prospective series of 77 patients assessed by FDG-PET 3 months after ASCT, and then every 6–12 months during follow-up (40). The duration of the response was longer when the PET scan was negative (27.6 months) than when it was positive (18 months, *p* = 0.05), whereas in patients with positive PET, SUVmax was inversely correlated with the duration of the response (*P* < 0.01).

However, the definition of CMR was not the same in these different clinical studies and a standardization of FDG-PET interpretation criteria should be done. Definition of cut-offs for FDG-PET positivity/negativity after therapy for MRD evaluation is currently underway. Preliminary results of a combined analysis of two European prospective trials have been presented by Zamagni et al. at the 2018 annual meeting of the ASH (41). In this joint analysis of 236 patients, attaining FL and bone marrow Deauville score <4 prior to maintenance therapy was the strongest independent predictor for prolonged PFS and OS and could be identified as the most representative cut-off value for PET negativity after therapy. Moreover, the CASSIOPET study is on-going, aiming to determine the best CMR threshold (mediastinal vs. hepatic background) on FDG-PET and try to establish the concordance between CMR and MRD negativity in the bone marrow (by flow cytometry or sequencing) to confirm the complementary role of functional imaging with modern biological tools for the detection of MRD inside and outside the bone marrow.

## Prognostic Value of FDG-PET at Relapse Setting

Although existing data are less available, FDG-PET seems to have also a prognostic impact at relapse workup. In a small series of 37 MM patients suspected of relapse after ASCT, it was shown that the absence of FL was a favorable prognostic factor for time to progression (TTP) and OS (42). The presence of more than 10 FL correlated with a shorter TTP and OS whilst a high SUV_max_ and the presence of EMD resulted in a longer TTP.

More recently, in a retrospective series of 40 confirmed relapsed patients, Nantes' group have described that the presence of at least 6 FL in the peripheral skeleton was an independent pejorative prognostic factor on both the PFS and the OS by multivariate analysis (43). Moreover, a high SUV_max_ (>15.9) was an independent negative prognostic factor on the PFS as was a high TLG of the hottest lesion (>98.1 g). Interestingly, 15% of the patients were FDG-PET positive without re-ascending the monoclonal peak and no change in the level of serum free-light chains.

Finally, scarce data on the value of FDG-PET before or after allo-SCT are available but two retrospective studies of heavily pre-treated MM patients showed FDG-PET results prior to and after allo-SCT were strongly associated with the outcome (44, 45).

## New PET Tracers

It has been recently reported in a 227 patients study with an initial diagnosis of symptomatic MM a FDG-PET negativity rate of 11% (13). It was found in this subgroup of patients a low expression of the hexokinase 2 gene (which catalyzes the first step of glycolysis) and consequently a FDG trapping in the cells. Indeed, for these patients FDG-PET is not an appropriate tool to evaluate MRD. Development of nuclear medicine offer new perspectives for MM imaging and other PET tracers, preliminarily investigated in limited series of MM patients, targeting other metabolic pathways or plasma cell receptors, could be potentially more sensitive and specific than FDG.

11C-Methionine, which uptake reflects the increased protein synthesis of malignant cells seems to correlate well with bone marrow infiltration and could be more sensitive than FDG to detect intra- and extra-medullary MM lesions (46).

Choline is a lipid PET tracer clinically used for the evaluation of relapse of prostate cancer. This tracer labeled with C^11^ was proposed years ago in a preliminary study in comparison to FDG on 10 patients affected by symptomatic MM (47) and showed Choline would reveal more lesions. Another study on the comparison of FDG and ^18^F-Choline presented similar results on 21 patients with symptomatic MM (48). Then it seems that Choline (either C^11^- or ^18^F-) has a better detection rate as compared to FDG in MM patients at staging. However, unfavorable physiological biodistribution (increased background of the liver parenchyma and of the bone marrow) is a limitation.

Pilot study comparing other lipid tracer (^11^C-Acetate) and FDG at diagnosis of symptomatic MM also showed acetate would reveal more lesions (49).

Another new and potentially interesting tracer is CXCR4. C – X – C chemokine receptor 4 (CXCR4) is a G-protein-coupled chemokine receptor family implicated in the process of cell migration as well as in the homing process of hematopoietic stem cells to the bone marrow, angiogenesis and cell proliferation.

In multiple myeloma, CXCR4 expression is associated to disease progression and poor prognosis (50). Most experience with CXCR4-directed PET imaging has been gained in MM and around two thirds of patients could overexpress the receptor on the myeloma cell surface.

^68^Ga-Pentixafor, that targets CXCR4 is a promising PET ligand (51) especially as potential target for myeloma specific treatment (for CXCR4-positive tumors) in a theranostic approach with preliminary encouraging results with good tolerance of the treatment, high initial response rates in advanced-stage MM cases (52). However, it has been reported that, in a non-negligible number of cases, FDG provided better detectability so further studies would be important to clarify this aspect (53). Moreover, receptor expression seems to be a dynamic process that could be highly influenced by preceding or concomitant chemotherapy (53).

^18^F-fludarabine (54) and immuno-PET targeting CD138 (55) and CD38 (56, 57) also showed promising results in preclinical models.

However, pending issues with these new tracers are willingness, inter-patient tumor heterogeneity for specific targets and the lack of prognostic data reported.

## Conclusion

FDG-PET is a powerful diagnostic tool for the detection of medullary and extra-medullary disease at the initial diagnosis of symptomatic MM with a pejorative prognostic value for the presence of EMD. Moreover, FDG-PET is the reference imaging technique to assess therapeutic response of symptomatic MM, evaluation being available much earlier than by MRI. The negativity of pre-ASCT FDG-PET is a favorable prognostic factor and the positivity of FDG-PET after ASCT, especially in patients with complete biological response, is an independent pejorative prognostic factor. The negativity of FDG-PET, intramedullary flow cytometry, and the ratio of serum free light chains would make it possible to define an optimal complete response (eradication of monoclonal plasma cells in all compartments). Ongoing prospective trials will try to establish the concordance between CMR and MRD negativity in the bone marrow to confirm the complementary role of functional imaging with modern biological tools for the detection of MRD inside and outside the bone marrow. We recommend to perform FDG-PET at initial work-up and after therapy (before maintenance) for detection of EMD, for patients with oligo/non-secretory MM and if a MRD assessment is performed. At relapse it is probably the best imaging technique to differentiate active disease from morphological scars and remodeling. Other PET tracers may also show interest in FDG-negative patients but should be evaluated in prospective clinical trials.

## Author Contributions

BJ and FK-B wrote the paper. CB, TC, CT, CN, EZ, LB, A-VM, MC, PM, and CB-M critically revised and improved the paper.

### Conflict of Interest Statement

The authors declare that the research was conducted in the absence of any commercial or financial relationships that could be construed as a potential conflict of interest.
